# HIV-1 predisposed to acquiring resistance to maraviroc (MVC) and other CCR5 antagonists *in vitro *has an inherent, low-level ability to utilize MVC-bound CCR5 for entry

**DOI:** 10.1186/1742-4690-8-89

**Published:** 2011-11-07

**Authors:** Michael Roche, Martin R Jakobsen, Anne Ellett, Hamid Salimiseyedabad, Becky Jubb, Mike Westby, Benhur Lee, Sharon R Lewin, Melissa J Churchill, Paul R Gorry

**Affiliations:** 1 Center for Virology, Burnet Institute, Melbourne, Victoria, Australia; 2 Department of Medicine, Monash University, Melbourne, Victoria, Australia; 3 Department of Medical Microbiology and Immunology, Aarhus University, Denmark; 4 Pfizer Global Research and Development, Sandwich, UK; 5 Department of Microbiology, Immunology and Molecular Genetics, David Geffen School of Medicine, UCLA, Los Angeles, CA, USA; 6 Infectious Diseases Unit, Alfred Hospital, Melbourne, Victoria, Australia; 7 Department of Microbiology, Monash University, Melbourne, Victoria, Australia; 8 Department of Microbiology and Immunology, University of Melbourne, Parkville, Victoria, Australia

## Abstract

**Background:**

Maraviroc (MVC) and other CCR5 antagonists are HIV-1 entry inhibitors that bind to- and alter the conformation of CCR5, such that CCR5 is no longer recognized by the viral gp120 envelope (Env) glycoproteins. Resistance to CCR5 antagonists results from HIV-1 Env acquiring the ability to utilize the drug-bound conformation of CCR5. Selecting for HIV-1 resistance to CCR5-antagonists *in vitro *is relatively difficult. However, the CCR5-using CC1/85 strain appears to be uniquely predisposed to acquiring resistance to several CCR5 antagonists *in vitro *including MVC, vicriviroc and AD101.

**Findings:**

Here, we show that Env derived from the parental CC1/85 strain is inherently capable of a low affinity interaction with MVC-bound CCR5. However, this phenotype was only revealed in 293-Affinofile cells and NP2-CD4/CCR5 cells that express very high levels of CCR5, and was masked in TZM-bl, JC53 and U87-CD4/CCR5 cells as well as PBMC, which express comparatively lower levels of CCR5 and which are more commonly used to detect resistance to CCR5 antagonists.

**Conclusions:**

Env derived from the CC1/85 strain of HIV-1 is inherently capable of a low-affinity interaction with MVC-bound CCR5, which helps explain the relative ease in which CC1/85 can acquire resistance to CCR5 antagonists *in vitro*. The detection of similar phenotypes in patients may identify those who could be at higher risk of virological failure on MVC.

## Introduction

Human immunodeficiency virus type 1 (HIV-1) entry is initiated by the interaction of the viral gp120 envelope (Env) glycoproteins with cellular CD4 and a coreceptor, either CCR5 or CXCR4 [1]. Maraviroc (MVC) and other CCR5-antagonists such as vicriviroc (VVC, also known as SCH-D), AD101 (a preclinical precursor of VVC), and aplaviroc (APL) are HIV-1 entry inhibitors that bind to- and alter the conformation of CCR5, such that CCR5 is no longer recognized by gp120 [1]. Thus, CCR5-antagonists are allosteric inhibitors of HIV-1 entry [2-4]. MVC has been approved for use in treatment-experienced and antiretroviral therapy (ART)-naïve HIV-1-infected adults who have no evidence of CXCR4-using virus in plasma [5]. As with other antiretrovirals, treatment with CCR5-antagonists can result in drug resistance leading to virological rebound. Although virological failure can arise from the emergence of CXCR4-using HIV-1 strains that were present at very low levels prior to initiation of a CCR5-antagonist [6], genuine resistance to CCR5-antagonists results from adaptive alterations in gp120 enabling recognition of the drug-bound conformation of CCR5 [7-15].

Being allosteric inhibitors of virus entry, resistance to CCR5-antagonists is evident by plateaus in virus inhibition curves below 100% inhibition [16]. The magnitude of the reduction in plateau height can be quantified as the maximal percent inhibition (MPI), which reflects the ability of HIV-1 gp120 to recognize the drug bound conformation of CCR5. For example, MPIs can be high (> 80%) [15] signifying a relatively inefficient ability of gp120 to utilize the drug-bound conformation of CCR5, or low (< 20%) [13] signifying relatively efficient utilization of drug-bound CCR5. However, MPIs can be influenced by differences in the level of CCR5 expression on target cell populations [9,11,12]. Generally, in cell lines, there is an inverse relationship between the MPI achieved by a given virus with resistance to a CCR5-antagonist, and the level of CCR5 expression. Clinically, MPIs of HIV-1 have been reported using the PhenoSense™ Entry assay [16], which uses the U87-CD4/CCR5 cell line. These cells express comparatively lower levels of CCR5 than other commonly used indicator cells such as TZM-bl, JC53 and NP2-CD4/CCR5 cells [12] and therefore, are likely to provide a relatively conservative measure of resistance to CCR5-antagonists. Consistent with this view, results from the clinical trials of MVC in treatment-experienced subjects (MOTIVATE) showed that most MVC-resistant viruses in subjects failing therapy had relatively high MPIs within the range of 80-95%, when tested using the PhenoSense™ Entry assay ([15] and references within).

The *in vitro *generation and characterization of HIV-1 variants with resistance to antiretroviral drugs is vital for elucidating resistance mechanisms. However, selecting for HIV-1 resistance to CCR5-antagonists is relatively difficult [16]. One particular HIV-1 strain, CC1/85 [17], has been used in a number of independent studies for the *in vitro *generation of HIV-1 resistance to different CCR5-antagonists including MVC, VVC and AD101 (for example, [16,18-20]). In fact, the published *in vitro *CCR5-antagonist resistance studies are heavily biased towards the characterization of resistant variants derived from CC1/85. The CC1/85 strain of HIV-1 may therefore be predisposed to acquiring resistance to CCR5- antagonists *in vitro*. Here, we sought to elucidate the phenotypic features of CC1/85 that underlie this predisposition. A better understanding of these mechanisms has the potential to identify subjects with increased risk of developing resistance to MVC and other CCR5-antagonists.

## Methods

MVC-Sens and MVC-Res plasmids contain the *env *gene of CC1/85 virus and a derivative with MVC-resistance, respectively, cloned into the pSVIII-Env expression vector [15,16]. Single-round luciferase reporter viruses pseudotyped with MVC-Senv or MVC-Res Envs, or with the CCR5-using (R5) YU2, JRCSF, NB6-C3 or NB8-C4 Envs as controls were produced as described previously [15]. The characterization and maintenance of TZM-bl, JC53, U87-CD4/CCR5, NP2-CD4/CCR5 and the dually CD4- and CCR5-inducible 293-Affinofile cells, and the preparation of peripheral blood mononuclear cells (PBMC) has been described previously [15,21]. Maraviroc resistance assays were conducted using Env-pseudotyped luciferase reporter viruses, or replication competent viruses carrying MVC-Res or MVC-Sens *env *genes, as described previously [15,16]. For experiments using 293-Affinofile cells, populations expressing CD4 together with different levels of CCR5 ranging from relatively low to high were generated by inducing the cells with 2.5 or 5 ng per ml of minocycline and either 15.6, 31.2, 62.5, 125, 250, 500, 1000 or 2000 nM of ponasterone A, as described previously [21]. Alterations in drug sensitivity were assessed by reductions in the MPI as described previously [15,16].

## Results

**Figure 1:**
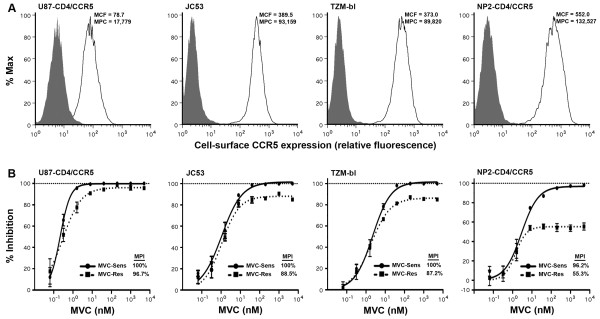
**Profiles of MVC resistance in cell lines expressing different levels of CCR5**. (A) U87-CD4/CCR5, JC53. TZM-bl and NP2-CD4/CCR5 cells were stained for cell-surface CCR5 expression, which was quantified by qFACS as described previously [21]. (B) Luciferase reporter viruses pseudotyped with MVC-Sens or MVC-Res Envs were used to infect the different cell lines in the presence of increasing concentrations of MVC as described in the Methods, and virus inhibition curves were generated and used to calculate the MPI as described previously [15]. The CCR5 cell surface staining data is representative of 3 independent experiments. The virus inhibition data are means of triplicates, and are derived from 3 independent experiments. Error bars represent standard error. MCF, mean cell fluorescence; MPC, molecules per cell; MPI, maximal percent inhibition.

**Figure 2:**
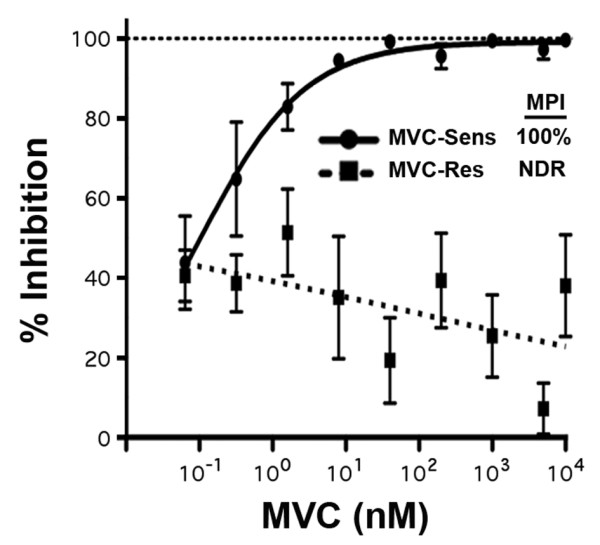
**Profiles of MVC resistance in primary peripheral blood mononuclear cells**. Luciferase reporter viruses pseudotyped with MVC-Sens or MVC-Res Envs were used to infect PHA-activated, IL-2-stimulated PBMC in the presence of increasing concentrations of MVC as described in the Methods, and virus inhibition curves were generated and used to calculate the MPI as described previously [15]. The virus inhibition data are means of triplicates, and are derived from 3 independent experiments using cells from different donors. Error bars represent standard error. MPI, maximal percent inhibition; NDR, no dose response.

As part of our ongoing studies of HIV-1 resistance to MVC and other CCR5-antagonists [15], in particular the influence of cell-surface CCR5 levels on the ability of resistant viruses to recognize the drug-bound conformation of CCR5, we first conducted MVC resistance assays for MVC-Res and MVC-Sens Envs in four different cell lines that express varying levels of CCR5, as well as PBMC. The cell lines included U87-CD4/CCR5 cells which express comparatively low-levels of CCR5, TZM-bl and JC53 cells which express comparatively moderate levels of CCR5, and NP2-CD4/CCR5 cells which express comparatively high levels of CCR5 (Figure 1A). The MPIs for MVC-Res Env ranged from approximately 55% in NP2-CD4/CCR5 cells to as high as 97% in U87-CD4/CCR5 cells (Figure 1B). Therefore, consistent with previous studies of VVC- and APL-resistant HIV-1 [11,12], we observed a close inverse relationship between CCR5 expression levels on cell lines and the magnitude of the MPI for MVC-Res Env. However, the most pronounced phenotypic resistance to MVC was in PBMC (Figure 2), which express much lower levels of CCR5, typically in the order of 5,000 to 12,000 molecules of CCR5 per CD4+/CCR5+ T-lymphocyte depending on the donor [22]. These results are consistent with those of previous studies, which suggest that distinct forms of CCR5 may exist on primary cells and cell lines that have varying affinities for CCR5 antagonists [23]. Unexpectedly, while virus pseudotyped with MVC-Sens Env was completely inhibited by MVC in U87-CD4/CCR5, TZM-bl and JC53 cells (Figure 1B) and PBMC (Figure 2A), this virus was consistently incompletely inhibited in NP2-CD4/CCR5 cells, plateauing at approximately 96% inhibition at the highest concentrations of MVC (Figure 1B). Interestingly, incomplete inhibition of Env derived from CC1/85 to MVC and another CCR5 antagonist, SCH-C has also been noted in two previous studies using different experimental approaches [16,24]. In contrast to the incomplete inhibition of virus pseudotyped with MVC-Sens Env in NP2-CD4/CCR5 cells, viruses pseudotyped with control YU2, JRCSF, NB6-C3 and NB8-C4 Envs were completely inhibited by MVC in these cells with MPIs consistently at 100% (data not shown). Together, these results show that MVC-Sens Env maintains a MVC-sensitive phenotype in PBMC and cell lines expressing relatively low or moderate CCR5 levels, including U87-CD4/CCR5 cells that are used in the PhenoSense™ Entry assay, but displays a low-level of basal resistance to MVC in NP2-CD4/CCR5 cells that express considerably higher levels of CCR5.

**Figure 3:**
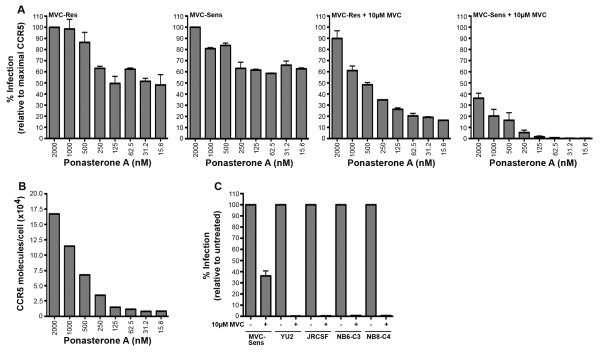
**Profiles of MVC resistance in 293-Affinofile cells induced to express different levels of CCR5**. (A) Luciferase reporter viruses pseudotyped with MVC-Res or MVC-Sens Envs were used to infect 293-Affinofile cell populations induced to express CD4 and increasing levels of CCR5 in the presence or absence of 10 μM MVC, and the percent infection was calculated relative to the levels of infection achieved in cells expressing the highest levels of CCR5 without drug, as described previously [15]. (B) The CCR5 induction levels in response to increasing concentrations of ponasterone A were quantified by qFACS as described previously [21]. (C) The inability of virus pseudotyped with MVC-Sens Env to be completely inhibited by MVC in 293-Affinofile cells expressing the highest CCR5 levels was compared to inhibition of virus pseudotyped with YU2, JRCSF, NB6-C3 or NB8-C4 Envs in these cells. The data shown are means of triplicates and are representative of 3 independent experiments. Error bars represent standard error.

To more precisely determine the relationship between CCR5 expression levels and the detection of this phenotype of MVC-Sens Env, we next performed MVC inhibition assays in 293-Affinofile cells where we could tightly control the induction of CCR5 in a uniform cell type. 293-Affinofile cells were induced to express CD4 together with eight different levels of CCR5 (ranging from approximately 8,000 to 167,000 molecules of CCR5 per cell) (Figure 3B) and subjected to entry assays with luciferase reporter viruses pseudotyped with MVC-Sens or MVC-Res Envs in the presence or absence of 10 μM MVC (Figure 3A). In the absence of drug, we observed a similar pattern of virus entry levels between MVC-Sens and MVC-Res Envs in all cell populations, indicating a similar CCR5-dependence profile. In the presence of drug, as expected [15], MVC-Res Env entered cells efficiently, particularly when cells were expressing high levels of CCR5. MVC-Sens Env entered cells expressing relatively high levels of CCR5 in the presence of drug, albeit less efficiently than MVC-Res Env, but was completely inhibited by MVC in cells expressing comparatively moderate and low-levels of CCR5. In contrast, luciferase reporter viruses pseudotyped with control YU2, JRCSF, NB6-C3 and NB8-C4 Envs were completely inhibited by MVC in 293-Affinofile cells expressing the highest levels of CCR5 (Figure 3C). Together, these results confirm the observations in NP2-CD4/CCR5 cells (Figure 1B) that MVC-Sens Env displays a low-level MVC-resistant phenotype that is revealed only when cells are expressing high levels of CCR5, but masked when cells are expressing moderate or relatively low-levels of CCR5.

**Figure 4:**
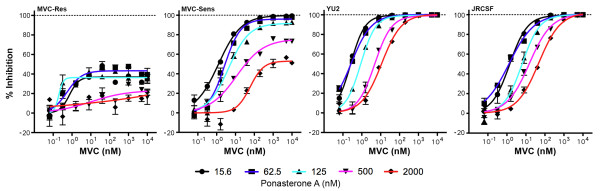
**Maraviroc inhibition curves in differentially-induced 293-Affinofile cell populations**. Luciferase reporter viruses pseudotyped with MVC-Sens, MVC-Res, YU2 or JRCSF Envs were used to infect 293-Affinofile cells induced with 2.5 ng per ml of minocycline together with increasing concentrations of ponasterone A, in the presence of increasing concentrations of MVC as described in the Methods, and virus inhibition curves were generated as described previously [15]. The data shown are means of triplicates and are representative of 3 independent experiments. Error bars represent standard error.

To determine whether this apparent low-level basal resistance could be due to an inherent ability of MVC-Sens Env to recognize drug-modified CCR5, we next produced virus inhibition curves for MVC-Sens and MVC-Res Envs in differentially induced 293-Affinofile cell populations (Figure 4). MVC-Res Env achieved plateaus of incomplete inhibition by MVC in all cell populations, with MPIs ranging from approximately 20 to 40% in a CCR5 concentration-dependent manner. MVC-Sens Env was completely inhibited by MVC in cells expressing low-levels of CCR5, but achieved plateaus of incomplete inhibition in cells expressing higher levels of CCR5, with MPIs ranging from approximately 53 to 100% in a CCR5 concentration-dependent manner. In contrast, viruses pseudotyped with control YU2 and JRCSF Envs achieved plateaus of 100% inhibition in all cell populations, despite a clear association between the MVC IC_50 _and CCR5 expression levels. Whilst the association between the MVC IC_50 _for YU2 and JRCSF and CCR5 expression levels suggests that more MVC is required to achieve complete inhibition of these viruses as CCR5 levels are increased, the plateaus of incomplete inhibition by MVC-Sens Env are consistent with the interpretation that it possesses the ability to interact with the drug-bound conformation of CCR5 [16]. However, the fact that this occurs only in cells expressing high levels of CCR5 supports the interpretation that this is a low affinity interaction [11]. Together, these results suggest that MVC-Sens Env can be distinguished from other R5 Envs by an inherent, yet relatively inefficient capability of recognizing the MVC-bound conformation of CCR5.

## Discussion and conclusions

Our results suggest that MVC-Sens Env, which was derived from the CC1/85 strain of HIV-1, has an inherent ability to recognize the MVC-modified conformation of CCR5. These results provide a plausible explanation as to why CC1/85, but not most other R5 HIV-1 strains, appears to be predisposed to acquiring resistance to MVC and other CCR5-antagonists *in vitro*, and why CC1/85 has been frequently used for this purpose in a number of independent studies [16,18-20]. The disclosure of the ability of MVC-Sens Env, but not other R5 Envs, to recognize drug-bound CCR5 in cell lines engineered to overexpress CCR5 distinguishes CC1/85 from other R5 viruses as being predisposed to develop a genuinely resistant profile when cultured in the presence of increasing concentrations of CCR5-antagonist. However, despite this predisposition, 17 virus passages and multiple Env mutations were required for CC1/85 to acquire resistance to MVC, compared to only 1 passage for this virus to acquire resistance to 3TC [16], suggesting that even CC1/85 has a relatively high genetic barrier to acquiring resistance to CCR5-antagonists.

The results of this study suggest that a similar baseline ability of HIV-1 to interact with drug-modified CCR5 may exist in certain subjects prior to commencing MVC or other CCR5-antagonists. To this end, in a longitudinal study of 21 ART-naïve subjects with HIV-1 subtype C, we have identified two subjects whose viruses exhibit plateaus of incomplete inhibition by MVC in NP2-CD4/CCR5 cells and 293-Affinofile cells expressing high levels of CCR5, but which are completely inhibited by MVC in cell lines expressing lower levels of CCR5, in a strikingly similar fashion as CC1/85 ([25], manuscript in preparation). In addition, retrospective analysis of 11 subjects who developed APL-resistance during the CCR100136 (EPIC) clinical trial of APL showed that baseline viruses of 8 individuals (73%) had some evidence of partial APL-resistance prior to therapy [8]. Further analysis of one of these baseline viruses confirmed that the Env glycoproteins had a low-affinity interaction with APL-bound CCR5 [12]. The clinical significance of viral variants with low-level basal recognition of drug-bound CCR5 in the setting of MVC and other CCR5-antagonist therapies remains to be determined by more extensive *in vivo *studies. To this end, we have shown that the likelihood of developing resistance to CCR5-antagonists *in vivo *is influenced also by the activity of the patient's optimized background therapy (B. Jubb and M. Westby, unpublished data). Nonetheless, our results suggest that certain individuals could be at increased risk of drug failure on MVC and other CCR5-antagonists due to predisposition for development of resistance.

One reason why U87-CD4/CCR5 cells are justifiably used by the PhenoSense™ Entry assay for the measurement of HIV-1 resistance to CCR5-antagonists is because the CCR5 expression levels on these cells more closely reflects CCR5 levels on primary CD4+ T-cells [22]. However, should future *in vivo *studies demonstrate that patients with baseline HIV-1 strains possessing inherent low-level ability to recognize drug-bound CCR5 are at greater risk of drug failure due to a predisposition to develop resistance, this phenotype is likely to be masked or only weakly exposed in U87-CD4/CCR5 cells. Pre-screening candidates for CCR5-antagonist therapy by a modified drug susceptibility assay using NP2-CD4/CCR5 cells or 293-Affinofile cells could potentially identify these individuals.

## Competing interests

BJ and MW are employed by Pfizer Global Research and Development. PRG and SRL are members of the ViiV Australia Scientific Advisory Board, and have received honoraria. SRL has received honoraria from ViiV for travel to conferences as well as for speaking at and Chairing ViiV-sponsored events. The other authors declare that they have no competing interests.

## Authors' contributions

MR, MRJ and PRG designed the experiments. MR, MRJ, AE and HS performed the experiments. BJ, MW and BL supplied critical reagents and helped interpret the results. SRL and MJC helped interpret the results. PRG and MJC supervised the project. MR and PRG wrote the manuscript. All authors helped edit the manuscript and have read and approved the final version.
